# Supplementary material for: 4D trajectory prediction and conflict detection in terminal areas based on an improved convolutional network
Source: PLoS One. 2025 Feb 14;20(2):e0317549. doi: 10.1371/journal.pone.0317549 (PMC11828371; doi:10.1371/journal.pone.0317549)
Supplement: Attachment — Submitted filename: Rebuttal_letter+PONE-D-24-28819+28082024.doc [file pone.0317549.s003.doc]

Dear Editors,

Thank you for allowing us to revise our paper, entitled “4D trajectory prediction and conflict detection in terminal areas based on an improved convolutional network” (Manuscript reference: PONE-D-24-28819). We would also like to extend our thanks to the reviewers. Their comments are very constructive and insightful for improving the quality of our paper. We have addressed all of the reviewers’ comments thoroughly. For the first modification, we use the blue background color to highlight the modified content. The details of our revision are provided below.

Sincerely yours.

Xin Ma, Master, Associate Professor

Linxin Zheng, Postgraduates

Xikang Lu, Postgraduates

(1)Nanjing University of Aeronautics and Astronautics, Nanjing, 211106, P. R. China;

(2)Civil Aviation Flight University of China，Guanghan, Sichuan, 618307, P. R. China.

**Responses to Reviewer 1**

#1. Expect the author to indicate the source of the training dataset.

**Response:** Thank you for your advice, this is a good question. This paper takes the ADS-B training dataset. We add the relevant description in the second section.

#2. The analysis of the images is not detailed enough.

**Response:** This is a very good suggestion. We supplement the substantive description of Fig. 5, Fig. 6, Fig. 7 and Fig.8 in the paper, and increase the analysis of the model and data.

#3. The conclusion is not comprehensive and precise enough, and the third point is the future work.

**Response:** Thank you very much for reviewers' comments. We refined the conclusion and gave a general description from the experimental data, the prediction model and the experimental results. And the third point is also changed to the description of the robustness of the article, so that the conclusion is more clear and comprehensive.

**Responses to Reviewer 2**

#1. 4D should be given its full name at the first appearance in abstract and paper.

**Response:** This is a very good suggestion. We have clearly given the full name at the first appearance in abstract and paper.

#2. Conclusions is too long, the discussion could be addressed seperately.

**Response:** Thank you very much for reviewers' comments. We refined the conclusion and gave a general description from the experimental data, the prediction model and the experimental results. And the third point is also changed to the description of the robustness of the article, so that the conclusion is more clear and comprehensive.

#3. What are the highlights in this paper？

**Response:** Thank you very much for reviewers' comments. The highlight of this paper is to create a new 4D trajectory prediction model named CNN-BiGRU, and use the trajectory distance detection function to realize the conflict assessment of the prediction results.

#4. The figures is not clear, the high quality should be improved.

**Response:** Thank you for your advice, this is a good question. We modify Fig.1, Fig.3 and Fig.4 respectively. After a series of fine optimization processing, the image quality has been significantly improved.

#5. The abstracts and conclusions should be improved with the highlights.

**Response:** Thank you very much for reviewers' comments. We refine and summarize the conclusion from three core aspects : firstly, we analyze the experimental data to ensure the solid foundation of the conclusion ; secondly, a new 4D trajectory prediction model is established to simulate the conflict detection function, and the conflict evaluation of the prediction results is realized. Finally, compared with other models, the accuracy of 4D trajectory prediction is improved.
